# Short- and Longterm Glycemic Control of Streptozotocin-Induced Diabetic Rats Using Different Insulin Preparations

**DOI:** 10.1371/journal.pone.0156346

**Published:** 2016-06-02

**Authors:** Gerd Luippold, Jessica Bedenik, Anke Voigt, Rolf Grempler

**Affiliations:** 1 Department of CardioMetabolic Diseases Research, Boehringer Ingelheim Pharma GmbH&Co.KG, Biberach an der Riss, Germany; 2 Department of Translational Medicine and Clinical Pharmacology, Boehringer Ingelheim Pharma GmbH&Co.KG, Biberach an der Riss, Germany; Communaute d\'Universites et d\'Etablissements Lille Nord de France, FRANCE

## Abstract

The chemical induction of diabetes with STZ has gained popularity because of the relative ease of rendering normal animals diabetic. Insulin substitution is required in STZ-rats in long-term studies to avoid ketoacidosis and consequently loss of animals. Aim of the present studies was to test different insulin preparations and different ways of administration in their ability to reduce blood glucose in STZ-induced diabetic rats. Single dosing of the long-acting insulin analogue glargine was able to dose-dependently reduce blood glucose over 4 h towards normoglycemia in STZ-treated rats. However, this effect was not sustained until 8 h post injection. A more sustained glucose-lowering effect was achieved using insulin-releasing implants. In STZ-rats, 1 insulin implant moderately lowered blood glucose levels 10 days after implantation, while 2 implants induced normoglycemia over the whole day. According to the glucose-lowering effect 1 as well as 2 insulin implants significantly reduced HbA_1c_ measured after 26 days of implantation. In line with the improved glucose homeostasis due to the implants, urinary glucose excretion was also blunted in STZ-treated rats with 2 implants. Since diabetic nephropathy is one of the complications of longterm diabetes, renal function was characterized in the STZ-rat model. Increases in creatinine clearance and urinary albumin excretion resemble early signs of diabetic nephropathy. These functional abnormalities of the kidney could clearly be corrected with insulin-releasing implants 27 days after implantation. The data show that diabetic STZ-rats respond to exogenous insulin with regard to glucose levels as well as kidney parameters and a suitable dose of insulin implants for glucose control was established. This animal model together with the insulin dosing regimen is suitable to address diabetes-induced early diabetic nephropathy and also to study combination therapies with insulin for the treatment of type 1 diabetes.

## Introduction

Diabetes mellitus is a metabolic disorder of multiple etiologies characterized by hyperglycemia resulting from defects in insulin secretion, insulin action or a combination of both. According to the International Diabetes Federation, 387 million people have diabetes and it is predicted to rise by 2035 to 592 million (www.idf.org). Type 2 diabetes mellitus is the most common form of diabetes, accounting for >90% of all cases in the western world. Type 1 diabetes mellitus (T1DM) is an autoimmune, metabolic disease characterized by a selective destruction of pancreatic β-cells by the immune system, leading to a loss of endogenous insulin production and insulin secretion [1–2].

Animal models with similarities to human T1DM include rodents that spontaneously develop diabetes, e.g. the non-obese diabetic (NOD) mouse or the bio breeding (BB) rat [3]. Adult rats injected with toxins, e.g. streptozotocin (STZ) or alloxan, which damage pancreatic β-cells, serve also as a predictable animal model of T1DM. STZ is a toxic glucose analogue that accumulates in pancreatic β-cells via uptake by the GLUT2 glucose transporter. STZ is split into its glucose and methylnitrosurea moiety. The latter is a powerful alkylating agent that induces multiple DNA strand breaks, causing β-cell damage [4].

The chemical induction of diabetes with STZ has gained popularity because of the relative ease of rendering normal animals diabetic. Due to the dose of STZ, non-ketonuric diabetes mellitus, where the animals can be maintained without insulin treatment for more than 100 days [5], could be separated from animals with a severe form of diabetes, suffering from diabetic ketoacidosis. These rats rarely survive a longer time period unless they are substituted by insulin [6].

Due to the absolute insulinopenia, insulin substitution is mandatory to obtain glucose control in T1DM patients. Accordingly, insulin substitution is also required in STZ-rats in longterm studies to avoid ketoacidosis. In a previous study, the glycemic control in STZ-diabetic rats on multiple regimens of different insulin preparations was investigated [7]. This study showed that protamine zinc insulin was problematic, causing regular episodes of hyperglycemia and hypoglycemia. A desirable profile of insulin in rodents includes reduction of hyperglycemia without large fluctuations or hypoglycemic episodes to mimic the desired profile of insulin in T1DM patients. The insulin analogue glargine is an extended-action biosynthetic human insulin (Aventis Pharma, Bad Soden, Germany). In T1DM patients, insulin glargine induces a constant concentration to time profile over 24 h without pronounced peaks, similar to the basal secretion of insulin. In rodents, insulin glargine has been shown to reduce blood glucose over a period of 2–3 h [8].

Aim of the present study was to test different insulin preparations in their ability to reduce blood glucose in STZ-diabetic rats. Since diabetic nephropathy is one of the complications of longterm diabetes, glycemic control has also been evaluated in an acute as well as subchronic setting in STZ-rats treated with insulin including the analysis of renal parameters. Objective was to qualify the subchronic STZ-model together with the insulin dosing regimen as a suitable model for the study of type 1 diabetes drugs in combination with insulin and possible longterm complications of diabetes-like nephropathy.

## Material and Methods

### Animals

All studies were performed in accordance with the German Law on the Protection of Animals and complied with the *Guide for Care and Use of Laboratory Animals* (Institute of Laboratory Animal Recources, 1996). The studies were approved by the responsible authority of Baden-Württemberg *(Regierungspräsidium Tübingen*, *Az*: *35/9185*.*81-8/09-032 and 35/9185*.*81-8/10-001)*. Male Sprague-Dawley rats (Crl:CD) with an age of 8–9 weeks (Charles River, Germany) were pretreated with a single intraperitoneal dose of 60 mg/kg streptozotocin (STZ) to induce experimental diabetes, which resembles a human type 1 diabetic condition. The manifestation of diabetes mellitus was verified by measuring the blood glucose 48 h following injection of STZ. Blood was collected from the tip of the tail. Only diabetic animals with a fed glucose levels of ≥15 mM were used for the various studies. Rats were housed in groups at controlled temperature and humidity conditions, with a 12-h light/dark cycle (lights out between 6:00 PM and 6:00 AM). The animals had free access to standard diet (Provimi Kliba, Suisse) and tap water. At the end of the experiments animals were sacrificed using intraperitoneal injections of pentobarbital (250 mg/kg).

### Insulin preparations

Different insulin preparations were used in the present studies, dependent on the desired pharmacological effect. First, insulin glargine which is an extended-action biosynthetic human insulin (Lantus^®^; Aventis Pharma, Bad Soden, Germany; product number 2F039A) that has been shown to reduce basal glucose in rodents [8] and second, insulin-releasing implants (Linplant^®^; Linshin, Scarborough, Canada; product number 4237) that release a basal dose of insulin (2 IU/24 hour/implant) for more than 40 days. The implants are formed from a mixture of human insulin and palmitic acid micro-crystals with a length of 7 mm and a diameter of 2 mm. These implants were inserted in the neck of the rats under short-acting anesthetic conditions (3 l/min of O_2_ + 2% isoflurane) according to the recommendations of the manufacturer.

### Analytical procedures

Blood glucose was measured with a glucometer (OneTouch Ultra; LifeScan, Milpitas, CA, Coefficient of Variation <5%) and albumin as well creatinine by an automated analyzer (Cobas Integra 400 plus, Roche Diagnostics, Indianapolis, IN). Using this analyzer glycated hemoglobin (HbA1c) in whole blood was also monitored. Ketone bodies in blood were measured with test strips (Precision Xtra; Abbott Diabetes Care, Alameda, CA).

### Insulin glargine

In an acute dose-response-study, blood glucose was followed over 8 h in non-fasted STZ-diabetic Sprague-Dawley rats. The rats had free access to food and tap water. A pre-dose blood sample was obtained by tail bleeding for randomization and blood glucose was measured 30, 60, 90 min and 2, 3, 4, 5, 6, 8 hours after administration of insulin glargine. At time point 0 min, the animals (n = 5 per group) were subcutaneously injected either with different doses of insulin glargine (1.5, 3, 4.5, 6 IU/animal) or isotonic NaCl.

### Insulin-releasing implants

In a first subchronic dose-response-study over 10 days, STZ-diabetic Sprague-Dawley rats were randomized according to the blood glucose levels (n = 5 per group). Different numbers of implants (0.5, 1, 1.5, 2 insulin implants) were inserted in the neck of the rats on day 0. Blood glucose profile (7:00/10:00 AM and 1:00/4:00/7:00 PM) was monitored on day 10. During the study, the rats had free access to food and tap water.

In a second subchronic study, STZ-diabetic Sprague-Dawley rats were randomized according to the blood glucose levels (n = 7 per group). Animals were inserted either no implant or 1 or 2 insulin-releasing implants in the neck on day 0. Blood glucose profile (7:00/10:00 AM and 1:00/4:00/7:00 PM) as well as blood ketone bodies were monitored on day 26 and HbA_1c_ on day 0 and 26. The rats had free access to food and tap water. On day 25, the animals were weighed and body weight development measured. Metabolic cage experiments were performed on day 27 over 3 h (7:00 AM to 10:00 AM) without access to food but unlimited access to tap water.

### Data Analysis

The blood glucose excursion profiles were used to integrate an area under the curve (AUC). Percent inhibition values for each insulin dose were generated from the AUC data after subtraction of the AUC of the respective control group. Urinary glucose excretion, creatinine clearance and urinary albumin to creatinine ratio were calculated according to standard equations. The data are presented as mean±SEM. Statistical comparisons were conducted by one-way or repeated measures two-way ANOVA followed by Bonferroni post-tests. A p value < 0.05 was considered to show a statistically significant difference.

## Results

### Acute dose-response study

**Fig 1:**
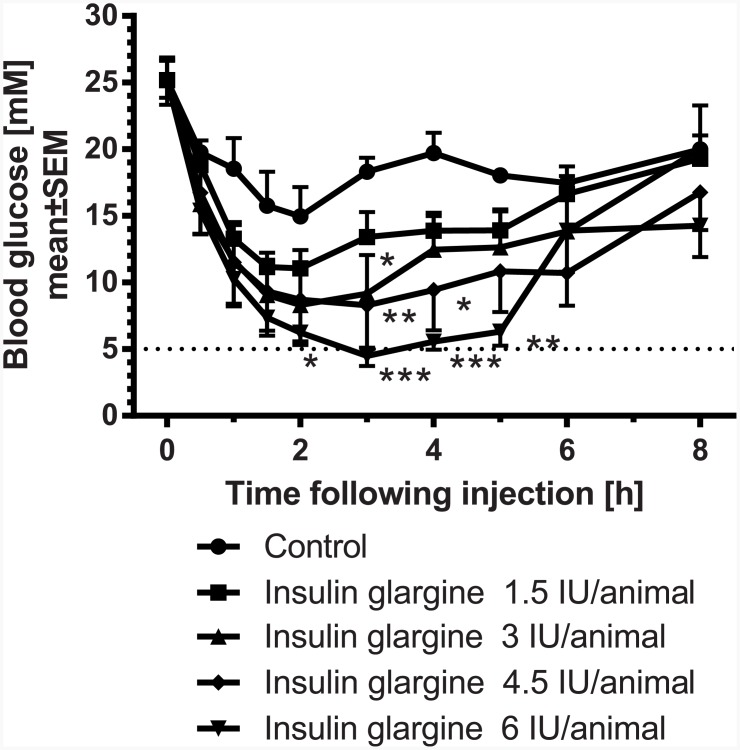
Dose-response curve of insulin glargine (1.5, 3, 4.5, 6 IU/animal) in STZ-diabetic rats over 8 h; normoglycemia is indicated by the dotted line. * p<0.05, ** p<0.01, *** p<0.001 vs. control.

**Fig 2:**
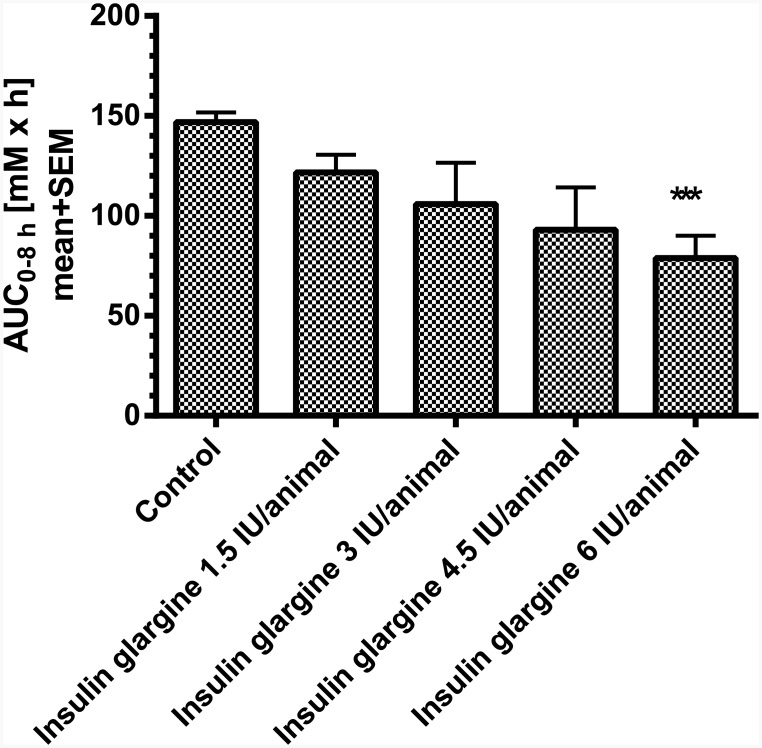
The calculated 8 h-blood glucose AUC (AUC_0-8h_) of STZ-rats (n = 5 per group). *** p<0.001 vs. control.

In an acute study, the blood glucose lowering effect of different doses of insulin glargine were evaluated. Therefore, non-fasted STZ-induced diabetic rats were randomized to pre-dose blood glucose levels (baseline: 25±2 mM) and subsequently injected with vehicle, 1.5 IU, 3 IU, 4.5 IU, and 6 IU insulin glargine per animal. Insulin was applied 2 weeks after induction of diabetes with STZ. Compared to controls insulin glargine dose-dependently decreased blood glucose (Fig 1). The maximum effect was observed with 6 IU, lowering the blood glucose to normoglycemia (~ 5 mM). In the different treatment groups blood glucose remained constant over approximately 5 h and returned to control levels at approx. 8 h post injection (Fig 1). The calculated 8 h-area under the curve (AUC_0-8h_) was significantly reduced by 45% following injection of 6 IU insulin glargine per animal (Fig 2).

### First subchronic dose-response study

**Fig 3:**
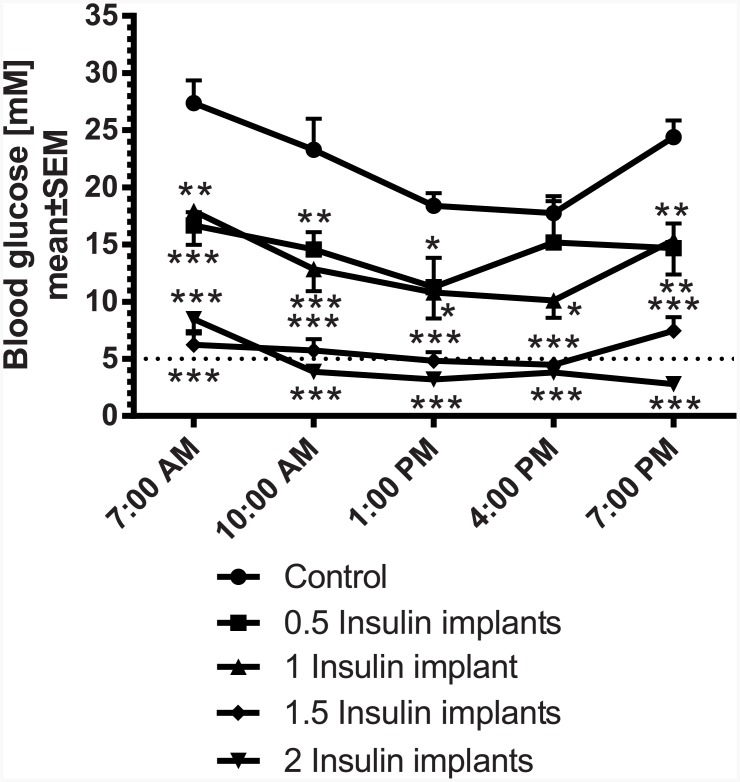
Blood glucose profile in STZ-diabetic rats 10 days after implantation of insulin implants (0.5, 1, 1.5, 2 implants/animal); normoglycemia is indicated by the dotted line. * p<0.05, ** p<0.01, *** p<0.001 vs. control.

**Fig 4:**
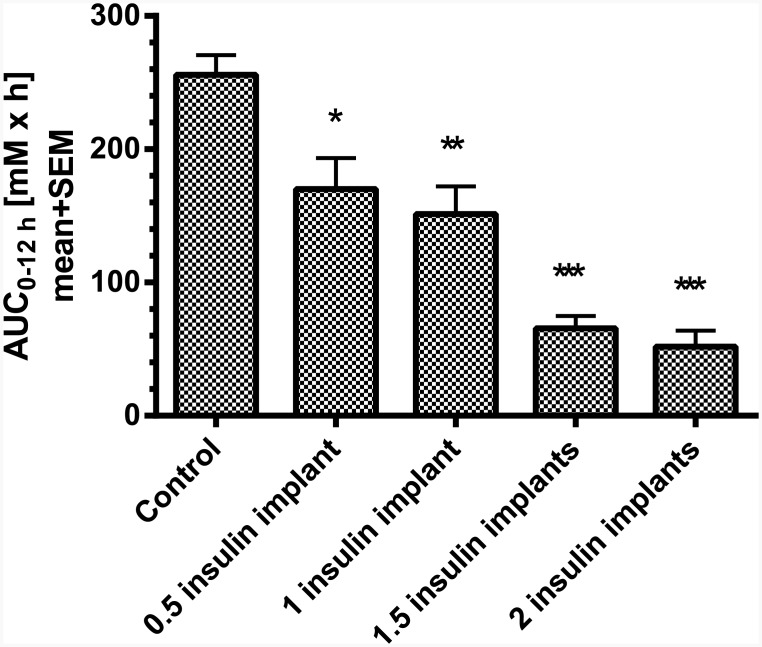
The calculated 12 h-blood glucose AUC (AUC_0-12h_) of STZ-rats (n = 5 per group). * p<0.05, ** p<0.01, *** p<0.001 vs. control.

In a first subchronic study over 10 days, the dose-dependent blood glucose lowering effect of different numbers of insulin releasing implants were analyzed. Therefore, non-fasted STZ-diabetic rats were implanted with 0.5, 1, 1.5 or 2 insulin implants after randomization to blood glucose (baseline: 25 mM±2 mM). Insulin implants were applied 2 weeks after induction of diabetes with STZ. On day 10 following implantation, the blood glucose profile was measured over 12 h (Fig 3). In the control group, blood glucose was approx. 27 mM at 7:00 AM, decreasing to approx. 20 mM at 4:00 PM and increasing thereafter to starting levels till 7:00 PM. In rats which received 0.5 or 1 implant, blood glucose levels at 7:00 AM were in the range of 17 mM and significantly lower than in the controls. Till 4:00 PM glucose decreased by 5 mM and tended to increase thereafter. Rats treated with 1.5 or 2 implants showed significantly lower blood glucose levels than in the controls in the range of 6–8 mM at 7:00 AM. With 1.5 implants blood glucose decreased to approx. 5 mM till 4:00 PM and tended to increase thereafter. Rats with 2 implants showed sustained normoglycemia from 10:00 AM to 7:00 PM. The calculated 12 h-blood glucose profile (AUC_0-12h_) of STZ-rats treated with 0.5 and 1 implant were similar (170.1±23.4 vs. 151.4±20.8 mM·h). Rats treated with 1.5 or 2 implants also showed similar reduction of glucose levels (65.6±9.1 vs. 51.9±11.9 mM·h). Each of the implants significantly lowered glucose AUC_0-12h_ compared to control STZ-rats (Fig 4) with 1.5 and 2 implants being most effective.

### Second subchronic study evaluating glucose homeostasis and kidney function

**Fig 5:**
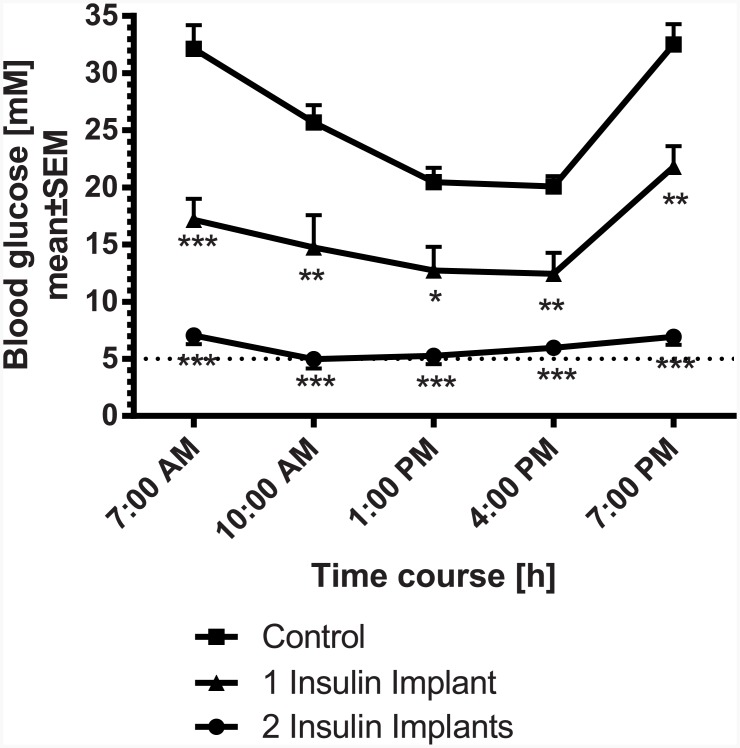
Blood glucose profile in STZ-diabetic rats 26 days after implantation of insulin implants (1 or 2 implants/animal; n = 7 per group); normoglycemia is indicated by the dotted line. * p<0.05, ** p<0.01, *** p<0.001 vs. control.

**Fig 6:**
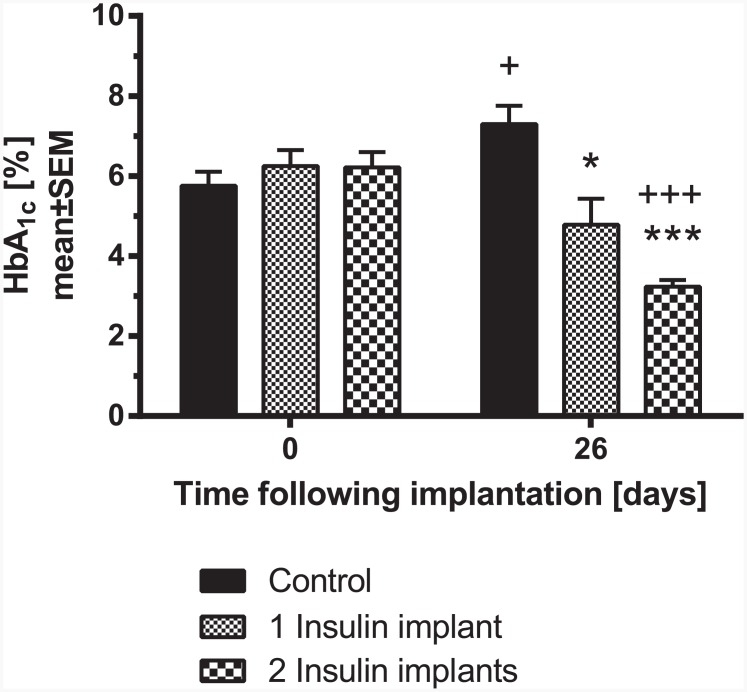
Blood HbA_1c_ in STZ-diabetic rats on day 0 and 26 days after implantation of insulin implants (1 or 2 implants/animal; n = 7 per group). * p<0.05, *** p<0.001 vs. control; ^+^ p<0.05, ^+++^ p<0.001 vs. day 0.

Following the results of the first subchronic study the effects of 1 and 2 insulin releasing implants were evaluated in STZ-diabetic rats over a longer time period. After randomization (baseline: 26±2 mM), apart from the controls, animals were implanted with either 1 or 2 insulin implants. Insulin implants were applied 2 weeks after induction of diabetes with STZ. On day 26, the blood glucose profile was measured over 12 h (Fig 5). At 7:00 AM, glucose was significantly lower for the rats treated with 1 insulin implant compared to control animals while 2 implants further decreased glucose to normoglycemic levels over 12 h. The increase in glucose between 4:00 PM and 7:00 PM, especially in the control groups and animals with 1 implant is due to the start of the dark cycle (6:00 PM) with increased food consumption of the rats. The calculated 12 h-blood glucose profile (AUC_0-12h_) of rats treated with 1 implant or 2 implants was significantly lower than for controls (178.3±24.6 or 69.6±6.1 vs. 295.6±11.3 mM·h, respectively). During the study, glucose homeostasis was additionally monitored by measuring HbA_1c_. HbA_1c_ levels significantly increased in the control group, indicating a worsening of glucose homeostasis over the study period (Fig 6). In contrast to the control group, 2 insulin implants significantly improved HbA_1c_ status over time when compared to day 0. A significant improvement on day 26 following implantation was observed in the rats treated with 1 insulin implant (-2.2% vs. control) and more prominent in animals treated with 2 insulin implants (-4.1% vs. control).

**Fig 7:**
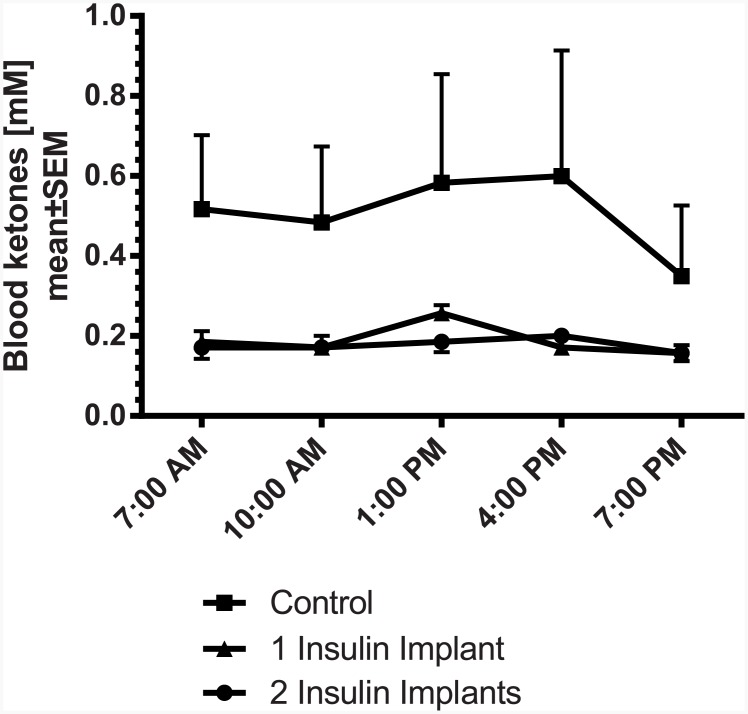
Blood ketone profile in STZ-diabetic rats 26 days after implantation of insulin implants (1 or 2 implants/animal; n = 7 per group). * p<0.05, ** p<0.01, *** p<0.001 vs. control.

Blood ketone bodies were measured as an indicator of the metabolic status of the rats. Blood ketones, monitored on day 26, tended to be lower in the rats with insulin implants compared to controls. However, variability in the control group was large (Fig 7). Body weight before implantation of the insulin implants was around 390 g per animal. During the 27 study days body weight slightly increased in the controls (Δ18.8 g±9.2). Body weight significantly increased in animals treated with 1 or 2 insulin implants compared to controls (Table 1), showing the improved metabolic condition of the rats. In the control group, urinary volume was 0.26 ml/min/kg and the urinary glucose excretion was calculated to 23.01 mg/min/kg, indicating the glucose-induced diuresis of the diabetic rats. Animals treated with 1 or 2 insulin implants had a significantly reduced urinary flow compared to controls (0.06 or 0.05 ml/min/kg, respectively) with a significantly attenuated glucose excretion of 1.82 mg/min/kg for 1 insulin implant and nearly no urinary glucose excretion with 0.01 mg/min/kg for 2 insulin implants. Since diabetic nephropathy is one of the complications of diabetes mellitus, estimation of renal function is mandatory in diabetic patients. Thereby, glomerular filtration rate (GFR) describes the flow rate of filtered fluid through the kidney. In this respect, creatinine clearance is the volume of blood plasma that is cleared of creatinine per unit time and is a useful measure for approximating the GFR. In the subchronic study, the creatinine clearance was calculated to 13.11 ml/min/kg in the STZ-treated control rats. Implantation of 1 or 2 insulin implants significantly reduced creatinine clearance to 8.55 and 8.97 ml/min/kg, respectively (Table 1). Microalbuminuria is one of the early signs of diabetic nephropathy. Therefore, measurement of urinary albumin (alb) to creatinine (crea) ratio is also indicated to assess renal function. In the control rats the ratio was 0.044 μg alb/g crea. Due to the insulin implants the value significantly decreased to 0.011 μg alb/g crea in the rats with 1 insulin implant and 0.006 μg alb/g crea in the rats with 2 insulin implants. Both values indicate an improvement of renal function with insulin treatment in the STZ-treated rats.

**Table 1:** Body weight gain (day 25 after implantation) and renal parameters in STZ-diabetic rats 27 days after implantation of insulin implants (1 or 2 implants/animal). * p<0.05, ** p<0.01, *** p<0.001 vs. control.

	Body weight gain [g]	Urinary volume [ml/min/kg]	Urinary glucose excretion [mg/min/kg]	Creatinine clearance [ml/min/kg]	Albumin/Creatinine ratio [μg/g]
**Control (n = 7)**	18.8±9.2	0.26±0.04	23.01±4.98	13.11±0.85	0.044±0.013
**1 Insulin implant (n = 7)**	45.6±4.9*	0.06±0.01***	1.82±0.94***	8.55±1.40*	0.011±0.002*
**2 Insulin implants (n = 7)**	79.5±4.8**	0.05±0.01***	0.01±0.01***	8.97±1.49*	0.006±0.001*

## Discussion

Aim of the present studies was to test different insulin preparations in their ability to reduce blood glucose in STZ-diabetic rats. Single dosing of the long-acting insulin analogue glargine was able to dose-dependently reduce blood glucose over 4 h towards normoglycemia in STZ-treated rats. A more pronounced glucose lowering was achieved using insulin-releasing implants. In STZ-rats, 1 insulin implant moderately lowered blood glucose levels 10 days after implantation, while 2 implants induced normoglycemia over the whole day. According to the glucose-lowering effect 1 as well as 2 insulin implants significantly reduced HbA_1c_ measured after 26 days of implantation. In line with the improved glucose homeostasis due to the implants, urinary glucose excretion was significantly reduced by 1 insulin implant and blunted by 2 insulin implants in STZ-treated rats, meaning that the renal glucose threshold was not reached in the latter case. Other renal parameters, like creatinine clearance and albuminuria appear elevated in STZ-treated rats after 26 days of hyperglycemia and were attenuated in rats supplemented with insulin implants. The data indicate that diabetic STZ-rats are suitable to address diabetes-induced renal dysfunction and that these rats respond to exogenous insulin when analyzing glucose levels as well as kidney parameters.

Dependent on the dose of STZ, different states of β-cell damage can be induced. In the present experiments the dose of STZ induced a type 1-like diabetes without loss of animals in the control groups. The insulin analogue glargine dose-dependently reduced glucose, reaching normoglycemia with the highest dose of 6 IU per animal over a period of 3–5 h, but clearly not over 24 h as shown in human T1DM patients [9]. The moderate efficacy in rats is in line with published experiments in rodents, in which insulin glargine reduced blood glucose over a period of 2–3 h [8]. A more stable glucose control without large fluctuations can be achieved by using insulin-releasing implants. In the present experiments in high-dose STZ-treated rats, 1 insulin-releasing implant per animal was able to adjust glucose to a moderate prandial hyperglycemia (around 15 mM) over the daytime, while 2 implants reduced glucose to normoglycemia in these rats. The efficacy of the insulin-releasing implants is preserved in our study at least for 26 days. This period has been confirmed by former experiments in rodents [8] and is in line with the manufacturer’s description. The product instructions state that at 45±5 days, the initial sustained release dose may occasionally (10%) appear less than optimal. Regarding convenience of implants compared to insulin injections, it is more effective and time-saving to implant rats once to influence hyperglycemia, than to inject insulin subcutaneously once or twice a day. Taken together, the insulin implants facilitate the management of diabetes mellitus in rats. One implant is sufficient to prevent loss of animals due to ketoacidosis but enables to use the animals as type 1-like diabetes model to study the efficacy of glucose lowering drugs e.g. in combination with insulin. In this respect, Kraynak et al. [10] demonstrated that STZ-induced DNA damage in rat kideys was detected up to 14 days after dosing with a return to background levels thereafter, indicating to consider a delay in the start of experimental therapies with the exception of insulin which is needed to keep the animals alive. In the present studies insulin was administered 2 weeks after induction of diabetes without any loss of animals.

Diabetes mellitus is the most prominent cause of chronic kidney diseases in the industrialized world. Pathophysiologically, diabetic nephropathy is characterized by structural alterations like glomerulosclerosis, mesangial cell expansion, podocyte loss and tubulointerstitial fibrosis. Functional aspects of this diabetic complication are changes in glomerular filtration rate and progressive albuminuria [11]. To understand the underlying pathogenesis and to test compounds interacting with pathways involved in diabetic nephropathy, animal models are urgently needed, that resemble pathological features observed in humans. In the present subchronic study, STZ-treated Sprague-Dawely rats show early functional signs of diabetic nephropathy 4 weeks after STZ injection. For instance, the STZ-rats show prominent urinary glucose excretion and elevated creatinine clearance resembling an early state of diabetic nephropathy, called diabetic hyperfiltration. This increase in glomerular filtration rate in diabetes might be a maladaptive response observed early in the course of diabetic kidney disease, which could eventually predispose to irreversible damage to nephrons and development of progressive renal disease [12]. The potential mechanisms leading to renal hyperfiltration in diabetes are not fully understood, although hypotheses have been proposed, including alterations in glomerular hemodynamic function and tubulo-glomerular feedback [13]. The first clinical sign of diabetic nephropathy is the microalbuminuria, which is based on the measurement of urinary albumin excretion [14]. In the STZ-treated rats albuminuria was also measurable, indicating renal dysfunction in rats. Insulin implants for 27 days clearly reduced urinary albumin excretion normalized for the glomerular filtration rate (ratio of albuminuria to creatinine), indicating a beneficial effect of glucose lowering on renal function. In this respect, Slaughter et al. [15] questioned the relevance of Sprague-Dawley rats as a model of renal injury when primarily evaluating structural alterations like glomerular injury or renal fibrosis. The present data, however, clearly showed that early functional parameters like creatinine clearance and albuminuria were elevated in STZ-induced Sprague-Dawley rats that could be controlled with insulin therapy. Obvious differences to the data of Slaughter et al. [15] are the doses of STZ (50 mg/kg versus 60 mg/kg in the present studies), the administration of one long-acting insulin implant to maintain glucose levels between 16 and 27 mM (no implant in the present study with glucose levels between 22 and 33 mM) and the additional manipulation of the rats (implanation of a catheter in the femoral artery).

In summary, HbA_1c_ was significantly increased over time together with a pronounced urinary flow and urinary glucose excretion in the STZ-rats. The insulin–releasing implants could clearly blunt these parameters. Regarding creatinine clearance and urinary albumin excretion as early signs of diabetic nephropathy, insulin implants were able to correct the functional abnormalities described in the STZ-rat model. Taken together, our results qualify the subchronic STZ-model together with the insulin dosing regimen as a suitable model for the study of drugs for the treatment of type 1 diabetes in combination with insulin and possible longterm complications of diabetes, i.e. early signs of diabetic nephropathy.
